# Are Children’s Externalizing and Internalizing Behaviours at 5 Years Predicted by Maternal Perinatal Depression Trajectory Profiles?

**DOI:** 10.3390/children12050535

**Published:** 2025-04-23

**Authors:** Stefan Kurbatfinski, Henry Ntanda, Jackson Mullin, Deborah Dewey, Brenda M. Y. Leung, Nicole Letourneau

**Affiliations:** 1 Department of Community Health Sciences, Cumming School of Medicine, University of Calgary, Calgary, AB T2N 1N4, Canada; stefan.kurbatfinski@ucalgary.ca; 2 Alberta Children’s Hospital Research Institute, Owerko Centre, Calgary, AB T2N 1N4, Canada; henry.ntanda@ucalgary.ca (H.N.); jackson.mullin@ucalgary.ca (J.M.); dmdewey@ucalgary.ca (D.D.); 3 Departments of Pediatrics and Community Health Sciences, Cumming School of Medicine, University of Calgary, Calgary, AB T2N 1N4, Canada; 4 Hotchkiss Brain Institute, University of Calgary, Calgary, AB T2N 1N4, Canada; 5 Faculty of Health Sciences, University of Lethbridge, Lethbridge, AB T1K 3M4, Canada; brenda.leung@uleth.ca; 6 Departments of Pediatrics, Psychiatry and Community Health Sciences, Faculty of Nursing, Cumming School of Medicine, Alberta Children’s Hospital Research Institute, Owerko Centre, University of Calgary, Calgary, AB T2N 1N4, Canada

**Keywords:** maternal depressive symptoms, child mental health, internalizing and externalizing problems, latent profile analysis, the APrON Study

## Abstract

**Background/Objectives:** Mothers’ depressive symptoms are associated with their children’s internalizing and externalizing behavioural problems. Because mothers’ depressive symptoms can vary across the prenatal and postnatal periods, considering their trajectories is important when predicting children’s behavioural problems. The purposes of this study were to: (1) identify profiles of mothers characterized by their prenatal and postnatal (up to 3 years postpartum) depressive symptom trajectories and (2) examine the associations between maternal depressive symptom profile trajectories and preschool children’s internalizing and externalizing behavioural problems at 5 years of age. **Methods:** This study used data derived from the APrON Study. The Edinburgh Postnatal Depression Scale measured mothers’ depressive symptoms in early (<27 weeks) and late (≥27 weeks) pregnancy and at 3, 6, 12, 24, and 36 months postpartum. The Behavioural Assessment Scales for Children, 2nd Edition, quantified children’s internalizing and externalizing problems at approximately 60 months of age. Non-growth latent profile analysis determined the most suitable and parsimonious number of maternal depressive symptom profiles, and linear regression analysis quantified their associations with their 5-year-old-children’s behavioural problems. **Results:** A three-profile structure characterized maternal depressive symptom trajectories: minimal, subclinical, and high. Unadjusted (n = 704) and adjusted (n = 621) analyses showed that: 1) mothers’ subclinical and high depressive symptom profiles (*p* < 0.01) predicted children’s internalizing problems and 2) mothers’ subclinical depressive symptom profiles (*p* < 0.01) predicted externalizing problems. **Conclusions:** Maternal subclinical depressive symptoms were equally, if not more, important compared to high depressive symptoms in predicting children’s behavioural problems. Overlooking mothers with subclinical depressive symptoms could have implications for their children’s behavioural/mental health.

## 1. Introduction

Globally, children’s mental health concerns have increased at an alarming rate and been further intensified by the COVID-19 pandemic [1,2]. Mental health concerns in children manifest as internalizing (e.g., anxiety, depression, and somatization) and externalizing (e.g., aggression, hyperactivity, and attention problems) behavioural problems [3], with sex-dependent outcomes often observed [4,5,6,7,8]. Behavioural problems in children can undermine their quality of life [9,10], and without intervention, the adverse effects can persist across the lifespan [11,12,13]. Several factors have been linked to the etiology of children’s behavioural problems [14]. Of those, mothers’ depressive symptoms have been identified as a strong predictor of children’s behavioural problems [15] and have been linked to less responsive, sensitive, and nurturing parent–child interactions [16,17]. This is particularly important to consider because the COVID-19 pandemic also exacerbated the number of stressors that mothers experienced [18], increasing their risk of developing depressive symptoms [19]. Although fathers’ depressive symptoms can also influence children’s behavioural problems [20], congruent literature emphasizes the more significant impact of mothers’ depressive symptoms on their children’s behavioural problems [15,21], and they are therefore the focus of this study.

The early childhood period is characterized by a time of extensive changes to the brain and rapid cognitive, behavioural, and emotional development [22,23]. Because of this state of neural plasticity, preschool children (two-to-five-year-olds) are especially susceptible to the adverse impacts of mothers’ depressive symptoms and therefore an important group toward which to employ preventive efforts to reduce risk for behavioural problems [24]. Statistical approaches such as latent profile analysis can provide insight into the associations among depressive symptom severity and changes over time in mothers and their preschool children’s behavioural problems. This information may be used to inform timely and relevant interventions.

### 1.1. Mothers’ Depressive Symptoms and Their Children’s Behavioural Problems

Mothers’ depressive symptoms, both prenatally and postnatally, have been strongly correlated with their children’s behavioural problems, although findings are conflicting [25]. Prenatally, mothers’ depressive symptoms, which have been associated with greater prenatal levels of the stress hormone cortisol [17,26,27], have been theorized to negatively alter fetal brain development through fetal programming mechanisms [28]. The fetal programming theory posits that prenatal conditions govern fetal development in accordance with the environment children are expected to reside in; through this mechanism, stress, and the associated cortisol hormone, signal to the fetus that the external environment is anticipated to be stressful [28]. Consequently, fetal brain development in structures related to stress regulation such as the amygdala and hippocampus are altered, resulting in a dysregulated stress response among these individuals once born [29]. While theoretically adaptive in nature [30], realistically, these mechanisms predispose children to dysregulated stress responses, which are associated with an increased likelihood of preschool children developing behavioural problems [31].

Postnatally, mothers’ depressive symptoms have been strongly associated with their preschool children’s behavioural problems [15]. Preschool children may be at particular risk of the impacts of mothers’ depressive symptoms as this is a period of rapid development [24]. In utero, infancy, and during the preschool period, two important neurobiological processes occur at an elevated rate: synaptogenesis and synaptic pruning [32,33]. Synaptogenesis refers to the formation of synapses in the brain [32]. It begins in utero and continues throughout life, with the most rapid growth occurring within the first 2 to 3 years of life [32]. Conversely, synaptic pruning involves the elimination of synapses which are underutilized [33]. Both processes are influenced by the prenatal and early childhood environment and are associated with structural and functional brain alterations that have been associated with cognitive and behavioural outcomes [32,33]. Preschool children’s experiences of nurturant and positive parent–child interactions, characterized by sensitivity and responsiveness, are associated with children’s greater emotional regulation and more optimal stress reactivity, responsiveness, and brain development [34]. These experiences are also associated with an increased likelihood of secure attachment, defined as the child’s confidence that their caregiver will provide safety and comfort when needed [35,36,37,38]. Conversely, exposure to inconsistent, unresponsive, and insensitive parent–child interactions, which are usually more prominent among mothers experiencing depressive symptoms, is associated with children’s reduced emotional and stress regulation [34], less than optimal brain development [39], and insecure and disorganized attachment [40,41,42]. These experiences and outcomes also increase children’s risk of developing behavioural problems [34,38,43,44,45]. Despite a strong association between mothers’ depressive symptoms and their preschool children’s behavioural problems [15], little is known about the influence of the course of maternal depressive symptoms over time on preschool children’s behavioural problems. Such information could assist in informing the development of evidence-based interventions.

### 1.2. Latent Classes/Profiles of Mothers’ Depressive Symptoms: What Is Known?

While some studies examining the association between mothers’ depressive symptoms and children’s behavioural problems employ analytical cross-sectional study designs, longitudinal studies can be leveraged to determine the association between changes in maternal depressive symptoms over time and preschool children’s behavioural problems [15]. The timing of exposure to mothers’ depressive symptoms is known to influence children’s behavioural problems [46,47]. Latent profile or class modeling can help to describe temporal effects, as it uses observations from multiple time points to characterize subgroups (i.e., profiles) based on similar characteristics, such as depressive symptoms [48]. Findings from studies using latent class or profile analysis to model both maternal prenatal and postnatal depressive symptoms reveal different numbers of classes/profiles, usually distinguishing three (e.g., no, mild, and moderate symptoms) [49], four (e.g., low level, early postpartum, subclinical, and persistent) [50,51,52], or five (e.g., non-depressed, postnatal-only, postpartum, prenatal-only, and chronic) [53] distinct classes/profiles. Other studies have employed latent growth curve analysis to describe associations between specific growth patterns (e.g., increasing, decreasing, and intermittent) in mothers’ depressive symptoms and their children’s behavioural and developmental outcomes [54,55]. All studies typically identified at least three classes/profiles that reflect, to some degree, minimal, moderate, and high depressive symptomology. Additionally, preschool children’s behavioural and/or executive function problems were generally more prevalent when predicted by more severe, increasing, and/or chronic maternal depressive symptom classes/profiles [49,50,51,52,53,54,55]. Collectively, these findings emphasize the importance of considering the associations between mothers’ depressive symptoms measured over time on their preschool children’s behavioural problems, as opposed to a single time point.

### 1.3. Children’s Behavioural Problems and Relevant Factors to Their Etiology

Internalizing and externalizing problems in children reflect two distinct manifestations of mental health concerns [3]. Internalizing problems, such as anxiety, depression, and somatization, are often self-directed, related to a child’s psychological state, and more difficult to identify [56]. Conversely, externalizing problems, such as aggression, hyperactivity, and attention problems, are often outwardly directed and more easily detected [56]. While the behaviours associated with internalizing and externalizing problems are contrasting, they all exemplify nonoptimal self-regulation strategies [57,58]. These behaviours and strategies have been associated with lifelong lower success in training, educational, and employment settings [11], adverse physical health sequelae [12], and relational difficulties [13,59]. Therefore, effective and timely intervention in the preschool period is crucial to reduce and prevent the emergence of behavioural problems and a cascade of downstream adverse outcomes.

Behavioural problems demonstrate some specificity by sex [60,61,62]. Findings from some studies suggest that male children appear to be more susceptible to developing both internalizing and externalizing problems [4,5,6,7,8], potentially attributable to different conditions during pregnancy (e.g., higher levels of testosterone in male pregnancies) [63] or Y-linked genetic contributions [64,65]. Alternately, diagnostic bias may simply favour detection among male children [66,67]. However, findings from other studies suggest that female children are more likely to develop internalizing problems and male children externalizing problems [60,61,62]. While genetic factors can help to explain this sex variance [68], gender roles and expectations according to which females are expected to be obedient and males are expected to be strong could also contribute to observed sex-specific behavioural problems [69,70]. To better understand the influence of factors such as mothers’ depressive symptoms on children’s behavioural problems, children’s sex-assigned-at-birth should be considered.

In addition to child sex-assigned-at-birth and parent–child interactions, socioeconomic factors may also contribute to children’s behavioural problems. Lower income, educational attainment, and unemployment are all conducive to inequitable healthcare access and societal engagement, which increase preschool children’s risk of developing behavioural problems [14,71,72]. Not only this, but higher socioeconomic status may prevent suboptimal mental health among mothers [73], helping to prevent intergenerational impacts on their children [15]. Racialized children also experience barriers in accessing and receiving high-quality healthcare, which may interfere with their access to effective intervention and increase the risk of behavioural problems not only emerging, but also going untreated [74]. In addition, racialized children are more likely to experience unique vulnerabilities such as exposure to war, displacements, and immigration that may increase their risk of developing behavioural problems [75,76]. Nevertheless, findings from other studies suggest that white children are more likely to develop behavioural problems [14]. Preterm birth has also been associated with behavioural problems [77,78]. Mothers who are older tend to reside in conditions of greater stability and maturity, both of which contribute to more optimal parent–child interactions and better behavioural outcomes among children [79]. Lastly, greater levels of perceived social support among mothers are often associated with better behavioural outcomes in children due to the impacts of social support on maternal mental health [80,81]. These protective effects are observed as early as the prenatal period and can indirectly help to promote children’s cognitive development and ability [82] by enhancing mothers’ perceived capacity to engage positively throughout parent–child interactions [83]. Including these important sociodemographic variables in analyses that predict children’s behavioural problems is crucial to controlling for extraneous effects.

### 1.4. Purpose of the Study

Latent profile modeling has been used to identify distinct maternal depressive symptom profiles and to determine their association with children’s health outcomes [49,50,51,52,53], including behavioural problems. The purpose of this study was two-fold: (1) to identify distinct groupings of women defined by their prenatal (early and late pregnancy) and postnatal (up to three years of child age) depressive symptoms and (2) to examine the associations between maternal depressive symptom profiles and preschool children’s internalizing and externalizing behavioural problems at five years of age. It was hypothesized that: (1) at least three latent profiles would be identified, reflecting minimal, subclinical, and high depressive symptomologies and that (2) children whose mothers had high depressive symptom profiles would exhibit the most internalizing and externalizing problems, followed by those whose mothers displayed a subclinical depressive symptom profile, when compared children with mothers categorized under the minimal depressive symptom profile. If profiles predicted children’s behavioural problems, post hoc analyses would examine if mothers’ depressive symptoms predicted various behavioural problem subscales (i.e., hyperactivity, aggression, attention problems, anxiety, depression, and somatization).

## 2. Methods

This quantitative study was reported using the 16 items enumerated in the Guidelines for Reporting on Latent Trajectory Studies (Supplementary Materials, Table S1) [84]. This study was reviewed and approved by the University of Calgary Health Research Ethics Board (REB14-1702) and the University of Alberta Health Research Ethics 11 Biomedical Panel (Pro00002954). Participants provided written, informed consent prior to data collection, and ongoing consent was obtained at each subsequent data collection period.

### 2.1. Study Design and Sample

Data were derived from the APrON Study, a longitudinal cohort that was designed to examine the associations between prenatal and postnatal factors, including prenatal nutrition, parental mental health and social support, and children’s neurodevelopmental outcomes [85]. Mothers who were less than 27 weeks into gestation were eligible to participate in the study if they were 16 years of age or older, could speak and read English, could attend appointments at the Universities of Calgary and Alberta, and planned to reside in the region until at least 3 months postpartum [85]. Recruitment involved several strategies, including approaching pregnant women directly in ultrasound waiting rooms, distributing posters and pamphlets, word of mouth, and online platforms (i.e., media, Internet) [86]. Participants did not receive any compensation for their participation. More detailed information is published elsewhere [85].

### 2.2. Measures and Covariates

#### 2.2.1. Predictive Variable

The Edinburgh Postnatal Depression Scale (EPDS) quantified mothers’ depressive symptoms [87]. Using a 10-item, Likert-type scale, the EPDS is a commonly employed measure of depressive symptoms from the prenatal to postnatal period [88,89], producing total scores ranging from 0 to 30, where higher scores are indicative of more depressive symptoms [87]. A score of 13 is often deemed a cut-off for probable major depression [90]. The EPDS demonstrates high validity and reliability in quantifying mothers’ depressive symptoms [91,92]. For example, Cronbach’s alpha for internal reliability consistency estimates have ranged from 0.82 to 0.84 when measured across pregnancy [91], while sensitivity and specificity have been reported at 0.66 and 0.95 for a cut-off score of 13 or higher, respectively [88]. Mothers’ depressive symptoms were assessed at each trimester of pregnancy and 3, 6, 12, 24, and 36 months postpartum. In early pregnancy (<27 weeks), depressive symptoms were measured in the first and/or second trimester; for those with two data points, the highest value was used in the present study. Third-trimester depressive symptom data characterized the late pregnancy (≥27 weeks) period.

#### 2.2.2. Outcome Variable

Children’s internalizing and externalizing behavioural problems were measured at approximately 5 years of age using the parent report of the Behavior Assessment System for Children, 2nd Edition, (BASC-2) [93]. The BASC-2 was standardized in a general American population that included a clinical sample of children with various diagnoses, including depression, anxiety, attention-deficit hyperactivity disorder, and conduct problems [93,94]. The Internalizing Problems Composite includes the Anxiety, Depression, and Somatization subscales, whereas the Externalizing Problems Composite includes the Aggression, Hyperactivity, and Attention Problem subscales [93]. Raw scores on the subscales and composites are converted to T-scores with a mean of 50 and a standard deviation of 10, allowing for a standardized method of comparing children’s behavioural problems to their peers [93]. Scores can range from 0 to 90, with scores above 70 classified as clinically significant, from 60 to 69 as at-risk, and below 60 as low-to-no risk of behaviour problems [93]. The BASC-2 has demonstrated high validity and reliability in assessing children’s behavioural problems [93,94]. For example, the BASC-2 has been shown to accurately differentiate between children with and without attention-deficit hyperactivity disorder (i.e., area-under-the-curve score of 0.919) [95]. In addition, internal reliability consistency estimates are relatively high but differ based on the population (e.g., 0.75 to 0.88 versus 0.81 to 0.88 in general versus clinical samples, respectively [93]) and/or subscale (e.g., 0.831 versus 0.877 for the Hyperactivity versus Depression subscales, respectively [96]) under study.

#### 2.2.3. Covariates

Child sex-assigned-at-birth was obtained from parents but validated using birth records; if a discrepancy was present, birth record data were used. Gestational age and birth weight were also collected from delivery records. Education, income, and marital status were collected at enrolment. A single socioeconomic status score ranging from 0 to 3 was determined by summing the values of household total annual income, maternal educational attainment, and marital status based on the following dichotomies: (a) income (CAD $), <$70,000 (0) or ≥$70,000 (1); (b) maternal educational attainment, less than a university degree (0) or a university degree or above (1); and (c) marital status, not married (0) or married (1). Mothers’ and children’s ages were determined by calculating the difference between the 5-year data collection date and birth dates. Mothers’ perceived social support was measured when children were approximately 3 years of age using the 4-item Social Support Questionnaire (SSQ) derived from the Canadian Community Health Survey, which assesses for informational, emotional, affirmational, and instrumental aspects of social support ([97], p. 194). The SSQ uses a 5-point Likert scale (0 = none of the time, 4 = all of the time) and results in scores ranging from 0 to 16, where higher scores indicate greater perceived social support ([97], p. 194).

### 2.3. Statistical Analysis

Descriptive statistics including means, standard deviations, percentages, and ranges were used to summarize sample characteristics and demographics. Heteroscedasticity was assessed using the Breusch–Pagan approach, where significant results suggest that the variance of the error terms is not constant across independent variables [98]. The Shapiro–Wilk test was used to test for normality, with significant values indicating that normality was violated. Multicollinearity was assessed using variance inflation factors; variance inflation factors close to 1 indicated that multicollinearity was absent. Outliers were identified both through visual inspection and the use of normal quantile–quantile plots. Lastly, Cook’s distance check was used to determine if outliers were influential, with values equal to 1 or lower suggesting that outliers were not influential [99].

#### 2.3.1. Determining Maternal Depressive Symptom Trajectory Profiles

To identify the trajectories of mothers’ depressive symptoms across early and late pregnancy and at 3, 6, 12, 24, and 36 months postpartum, latent profile analysis was conducted using MPlus version 7.11 [100]. The analysis began with fitting a one-profile model, followed by an iterative process of fitting models with an increasing number of profiles to identify the most parsimonious solution. Model fit was evaluated by comparing several criteria across successive models, including the likelihood ratio statistic (L2), the Akaike Information Criterion (AIC), and the Bayesian Information Criterion (BIC), with lower values for each variable indicating better fit [101]. Additionally, entropy was calculated for each model to quantify the precision of classification, where higher values signified more accurate assignment of individuals to latent profiles [101]. To compare whether the addition of a profile improved model fit, the Vuong–Lo–Mendell–Rubin likelihood ratio test (VLMR-L2) was employed with a statistical threshold of α = 0.05 [102].

#### 2.3.2. Analyzing the Association Between Maternal Depression Profiles and Child Behaviour

Covariates were included in adjusted models based on theoretical relevance, as opposed to statistical, to control for any epi-phenomenological effects. General linear regression was used to analyze the association between maternal depressive symptom profiles and children’s internalizing and externalizing behaviours. Unadjusted and adjusted analyses were conducted. For adjusted analysis, which included child sex-assigned-at-birth, mothers’ social support, socioeconomic status, and maternal age as covariates, 83 complete cases were removed through listwise deletion due to missing data. The adjusted model considered was of the general form:

$${Behavioural\;problem}_{i}=\beta_{0}intercept+\beta_{1}{depression\;profiles}_{i}+\beta_{2}{sex}_{i}+\beta_{3}socioeconomic\;status+\beta_{4}gestational\;age+\beta_{5}social\;Support+\varepsilon_{i}$$

where (1) i refers to the *i^th^* observation; (2) $\beta_{0}$ is the intercept value; (3) $\beta_{1},\beta_{2},\beta_{3},\beta_{4},and\;\beta_{5}$ are the coefficients for the predictor variable (i.e., depressive symptom profile) and covariates (i.e., child sex-assigned-at-birth, socioeconomic status, gestational age at birth, and maternal social support at 3 years); and (4) $\varepsilon_{i}$ is the random error term, which was assumed to be normally distributed with a mean of 0 and variance of $\sigma^{2}$. If associations were observed, post hoc analyses using analysis of variance (ANOVA) were planned to examine significant differences on the clinical problems subscales of the BASC-2 externalizing (i.e., aggression, hyperactivity, and attention problems) and internalizing (i.e., anxiety, depression, and somatization) behavioural problems between identified maternal depressive symptom profiles [103].

## 3. Results

### 3.1. Sample Characteristics

Demographic characteristics for the sample (n = 704) used in unadjusted analyses are provided along with the number of missing observations for each covariate (Table 1). For adjusted analysis, complete cases (i.e., those with available data for all covariates) were available for 621 individuals. Most of the participating mothers were married (n = 687, 97.72%), reported total household annual incomes greater than $70,000 CAD (n = 598, 86.04%), and had attained a postsecondary degree (n = 534, 76.18%). Children’s mean age at delivery was 39.27 weeks (±1.59), and mean birthweight was 3377.99 g (±515.53). The proportions of female (49%) and male (51%) children were relatively similar.

### 3.2. Tests of Assumption

Findings from the Breusch–Pagan test suggested that heteroscedasticity was present for internalizing (*p* < 0.01) but not externalizing (*p* > 0.06) problems. Although the error terms for both internalizing (*p* < 0.04) and externalizing (*p* < 0.01) problems appeared to be non-normally distributed, general linear modeling was employed due to robustness to violations of the normality assumption, especially in large samples such as this one [104,105]. Further, general linear regression modeling is commonly employed in behavioural and health sciences even in the presence of non-normal distributions [106]. Variance inflation factor estimates were close to 1 for both internalizing and externalizing problems, suggesting that multicollinearity was not an issue. Lastly, outliers were present for either internalizing or externalizing behavioural problems according to visual inspection and testing use normal quantile–quantile plots; however, values obtained from Cook’s distance check were equal to or below 1, suggesting that the outliers were not influential.

### 3.3. Identifying Trajectory Profiles of Maternal Depressive Symptoms

Latent profile models using mothers’ depressive symptoms across early and late pregnancy and at 3, 6, 12, 24, and 36 months postpartum were estimated through one-to-four-profile models (Table 2). The Vuong–Lo–Mendell–Rubin likelihood ratio test indicated a significant difference between the one- and two-profile models and the two- and three-profile models, and no difference between the three- and four-profile models, suggesting that the three-profile model performed most effectively (Table 2). Because the four-profile model did not perform better than the three-profile model, higher-profile models were not tested. The likelihood ratio, Bayesian information criterion, and Akaike information criterion statistics of the three-profile model were lower than those of the one- and two-profile models, further confirming the improved model fit (Table 2). In addition, the entropy value for the three-profile model was higher (0.88) than for all other profile models, suggesting that the three-profile model had the highest precision in assigning individual cases to their appropriate profile (Table 2). An examination of the sample sizes of each latent profile in the three-profile model revealed that all profiles had a sufficient sample size, with the smallest profile (n = 65) constituting a meaningful profile composed of a sufficient number of participants (Table 1). Therefore, the three-profile model was accepted as the final model.

**Figure 1:**
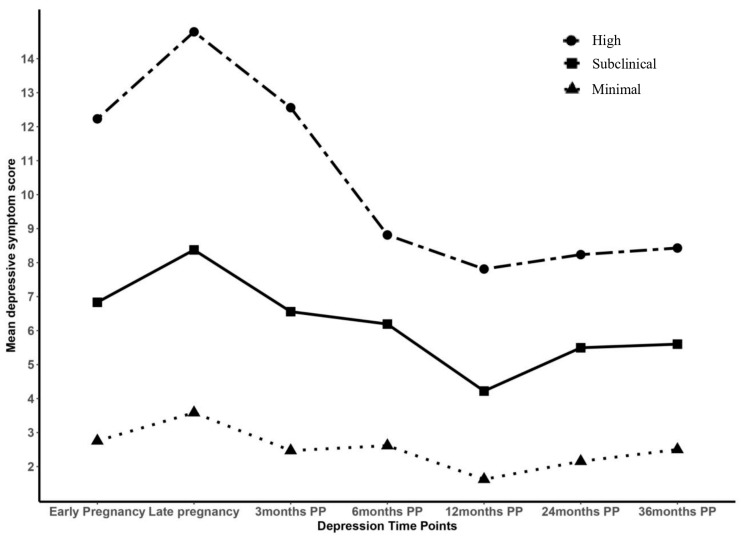
Maternal depressive symptom trajectory profiles according to a three-profile model (n = 704).

The three maternal depressive symptom trajectory profiles are illustrated (Figure 1). The first and largest trajectory profile consisted of women who reported “minimal depressive symptoms” (n = 361, 51.28%). The second trajectory consisted of women who reported “subclinical depressive symptoms” (n = 278, 39.49%). The third and smallest trajectory profile consisted of women who reported “high depressive symptoms” (n = 65, 9.23%). All three trajectories show an increase in depressive symptoms from early to late pregnancy, generally followed by a decrease until 12 months postpartum, though the decrease is sharpest for the high depressive symptom profile, with a small and gradual increase until 36 months postpartum (Figure 1). Overall, patterns are generally similar, except at 6 months postpartum (Figure 1).

### 3.4. Associations with Children’s Internalizing Behavioural Problems

The linear regression model indicated that the intercept represents the expected level of internalizing problems when the predictor variable (i.e., mothers’ depressive symptom profiles) was set to zero (Table 3). In unadjusted analysis, children of mothers in the subclinical and high depressive symptom profiles had a 3.73 T-score-unit (SE = 0.69, *p* < 0.01, 95% CI [2.39, 5.08]) and 3.78 T-score-unit (SE = 1.16, *p* < 0.01, 95% CI [1.51, 6.05]) increase in internalizing problems, respectively, relative to those whose mothers displayed a minimum depressive symptom profile. Post hoc ANOVAs revealed that while children’s internalizing problems differed between subclinical profiles and minimal depressive symptom profiles, and between high and minimal depressive symptom profiles, no difference was observed between subclinical and high depressive symptoms profiles. After adjusting for covariates, results were reduced but remained significant; children of mothers categorized under the subclinical and high depressive symptom profiles displayed a 3.01 T-score-unit (SE = 0.71, *p* < 0.01, 95% CI [1.40, 4.43]) and 2.81 T-score-unit (SE = 1.22, *p* < 0.03, 95% CI [0.39, 5.22]) increase in internalizing problems, respectively, relative to those whose mothers exhibited the minimum depressive symptom profile. Setting male children as the referent group, female sex-assigned-at-birth was not associated with children’s internalizing problems (estimate = 1.19, SE = 0.66, *p* > 0.05, 95% CI [−0.10, 2.50]). As mothers’ perceived social support (SE = 0.12, *p* < 0.01, 95% CI [−0.60, −0.10]) increased, children had a 0.35 T-score-unit decrease in internalizing problems. Gestational age (estimate = 0.03, SE = 0.21, *p* > 0.05, 95% CI [−0.38, 0.45]), maternal age (estimate = −0.06, SE = 0.08, *p* > 0.05, 95% CI [−0.24, 0.10]), and socioeconomic status (estimate = −0.67, SE = 0.54, *p* > 0.05, 95% CI [−1.73, 0.38]) were not associated with children’s internalizing problems.

### 3.5. Associations with Children’s Externalizing Behavioural Problems

The linear regression model indicates that the intercept represents the expected level of externalizing problems when the predictor variable (i.e., mothers’ depressive symptom profiles) was set to zero (Table 4). In unadjusted analysis, children of mothers in the subclinical profile group had a 2.42 T-score-unit (SE = 0.63, *p* < 0.01, 95% CI [1.18, 3.67]) increase in externalizing problems relative to those whose mothers who were in the minimum depressive symptoms profile group. High depressive symptom trajectories were not associated with children’s externalizing problems (estimate = 1.67, SE = 1.07, *p* > 0.05, 95% CI [0.43, 3.77]). Post hoc ANOVAs revealed no differences between the subclinical and high depressive symptoms profiles. After adjusting for covariates, results were reduced but remained significant; children of mothers in the subclinical depressive symptom profile group had a 2.20 T-score-unit (SE = 0.68, *p* < 0.01, 95% CI [0.86, 3.54]) increase in externalizing problems relative to those whose mothers were in the minimum depressive symptom profile group. High depressive symptom trajectories were not associated with children’s externalizing problems (estimate = 1.01, SE = 1.16, *p* > 0.05, 95% CI [−1.28, 3.30]). When holding all other variables constant, sex-specific outcomes were observed; male children had a 2.16 T-score-unit (SE = 0.63, *p* < 0.01, 95% CI [−3.41, −0.92]) increase in externalizing problems compared to female children, suggesting that they were more likely to display externalizing behavioural problems (Table 4). In addition, as mothers’ socioeconomic status increased, children exhibited a decrease of 1.63 T-score units (SE = 0.51, *p* < 0.01, 95% CI [−2.64, −0.62]) in externalizing problems. Mothers’ perceived social support (estimate = −0.06, SE = 0.12, *p* > 0.05, 95% CI [−0.30, 0.17]), gestational age (estimate = −0.08, SE = 0.20, *p* > 0.05, 95% CI [−0.48, 0.30]), and maternal age (estimate = 0.01, SE = 0.08, *p* > 0.05, 95% CI [−0.15, 0.17]) were not associated with children’s externalizing problems.

### 3.6. Post Hoc Exploration: Associations with Subscales of Behavioural Problems

Post hoc exploration consisted of examining if depressive symptom trajectory profiles were associated with subscales of behavioural problems (Table 5). In unadjusted models, all externalizing (i.e., hyperactivity, aggression, and attention problems) and internalizing (i.e., anxiety, depression, and somatization) problems were associated with the subclinical depressive symptom profile (*p* < 0.01), whereas only anxiety was associated with the high depressive symptom profile (Table 5). In the adjusted analysis, the findings remained significant except for somatization, which was no longer associated with the subclinical depressive symptom profile (*p* > 0.05). In the adjusted analysis, all three externalizing behavioural problems were associated with socioeconomic status and child sex-assigned-at-birth such that male children were more likely to display hyperactivity, aggression, and attention problems (*p* < 0.01; Table 5). Social support at 3 years of child age was associated with children’s attention problems and anxiety (Table 5). Gestational and maternal age were not associated with any behavioural problem subscales (Table 5).

## 4. Discussion

This study characterized three distinct profiles of maternal depressive symptoms measured prenatally and postpartum and examined their associations with their preschool children’s behavioural problems at 5 years of age. Latent profile analysis for mothers’ depressive symptom trajectories revealed that a three-profile structure was the most suitable model. Children of mothers experiencing subclinical levels of depressive symptoms exhibited more internalizing and externalizing behavioural problems compared to those whose mothers experienced minimal depressive symptoms, which also appeared clinically relevant. In contrast, the children of mothers with a high depressive symptom profile displayed only higher internalizing problems, again showing clinical relevance regarding children’s behavioural outcomes. Child sex-assigned-at-birth was also associated with externalizing problems only, such that male children were more likely to exhibit these types of behavioural problems, with clinical relevance. In the adjusted analysis, socioeconomic status and maternal perceived social support appeared to play a protective role in children’s externalizing and internalizing behavioural problems, respectively, though these findings were not as clinically relevant as others.

### 4.1. Mothers’ Perinatal Depressive Symptoms Are Best Reflected by a Three-Profile Structure

The three-profile structure of mothers’ depressive symptoms characterized by minimal, subclinical, and high depressive symptom profiles was deemed the most suitable and parsimonious model, parallelling the three-profile structure observed in Oh, Joung, Baek, and Yoo [49]’s study conducted in South Korea. Further, while findings from other studies observed more profiles, they generally described three profiles like those found in this study [50,51,52,53]. Characterized by prenatal and postnatal depressive symptoms, the depressive symptom trajectories we observed are consistent with expected biological and psychosocial mechanisms [107]. Indeed, findings from other studies indicate a greater prevalence of depressive symptoms as pregnancy progresses, as observed in this study [108]. While only hypothesized, differences in depressive symptom profiles could be attributed to differing levels of prenatal stress [109], which can exacerbate the experience of depressive symptoms [109]. Alternately, genetic susceptibility may place some mothers at greater risk of experiencing perinatal depression [110].

The research literature indicates that the first few months following birth (0 to 6 months) are marked by a sharp increase in cortisol and greater levels of stress, which decrease over time [109,111]. The decrease may be even more visible for mothers with high depressive symptoms, as comparatively greater levels of cortisol right after pregnancy are more visibly reduced over time [112]. Often, depressive symptoms have been observed to decrease within the first year postpartum [112]; however, after 6 months, levels appeared to stabilize among each depressive symptom profile and remained higher for mothers experiencing high depressive symptoms, a finding corroborating the existing literature [113]. From 12 to 36 months postpartum, all three profiles were characterized by a small increase in depressive symptoms. This could be attributable to the stress that mothers may experience returning to work [114], aligning with the ending of employment insurance received through maternity and parental benefits programs in Canada [115], which permit mothers to take up to 12 months off work after giving birth. Such an extended leave from work is more likely in higher-socioeconomic-status samples such as this one [114]. Concomitantly caring for children and attending to occupational responsibilities could also overburden mothers, especially when their partners are working, increasing their perceived stress and eventually leading to higher levels of depressive symptoms [116]. On the other hand, mothers may simply experience stress being apart from their child [116]. This slight increase in depressive symptoms among all three profile groups could also be attributable to children’s socioemotional growth [117]. As children reach 2-to-3 years of age, they become more autonomous, and their feelings and emotions can become challenging (e.g., tantrums) and difficult to manage, challenging mothers’ navigation of motherhood [117]. With this awareness, employers and family practitioners should support mothers in navigating these challenging time periods.

### 4.2. Subclinical and High Depressive Symptom Profiles and Children’s Behavioural Problems

Children of mothers in the subclinical and high depressive symptom profile groups exhibited more internalizing problems when compared to the minimal depressive symptom profile group, while the subclinical depressive symptom profile was only associated with children’s externalizing problems, generally supporting what was hypothesized. As the high depressive symptom profile was not associated with children’s externalizing problems, perhaps children of mothers who experience high levels of maternal depressive symptoms internalize their emotional dysregulation, rather than externalize it. This may be explained by the less-than-optimal limit-setting and dissatisfaction with parent–child interactions that highly depressed mothers may experience [118]. Nevertheless, our findings generally paralleled those from single-timepoint (analytical cross-sectional) and multiple-timepoint (longitudinal) studies [15]. This research provides additional evidence that mothers’ depressive symptoms are positively associated with behavioural problems in their children [15]. During pregnancy, it is posited that high levels of prenatal cortisol alter typical fetal development, leading to worsened behavioural outcomes postpartum [28]. Maternal depressive symptoms and the associated release of cortisol may also impact fetal development indirectly through cascading effects on different biomarkers, such as cytokines (immune system) or serotonin (neurotransmitter), which are known to impact children’s development [28].

In the postpartum period, mothers who experience depressive symptoms are likely to engage in unresponsive, inconsistent, and insensitive parent–child interactions [119]. These are associated with mothers’ unresolved depressive symptoms and, in turn, linked to children’s insecure attachment [120], reduced capacity to self-regulate, and less-than-optimal physiological response to external stressors [121]. The first year of life is especially important to attachment development [122,123], and the first three years are associated with significant changes in brain architecture through synaptogenesis and synaptic pruning [124]. Therefore, suboptimal child attachment and parent–child interaction quality could substantially affect children’s neurodevelopment and lead to the emergence of behavioural problems in preschool children [34,39]. Interventions that improve mothers’ sensitivity and responsiveness in parent–child interactions, but not their depressive symptoms, have been found to be associated with positive effects on children’s cognitive and social-emotional outcomes [125,126,127]. Overall, findings from this study affirm that the perinatal period, infancy, and early childhood are important times in which mothers’ depressive symptoms can significantly impact their children’s behavioural outcomes.

Although high levels of maternal depressive symptoms were associated with more internalizing problems among children, the impact on children’s behavioural problems was not statistically significantly different compared to mothers with subclinical levels of depressive symptoms; this is an important finding, as it shows that subclinical levels of depressive symptoms can still influence children’s behavioural outcomes. Further, it is consistent with the findings of a recent study that reported that parental depressive symptoms below the clinical cut-off remained a significant predictor of child behavioural problems [128]. It is possible that mothers who may not display levels of depression indicative of major depression could still experience symptoms that undermine parent–child interaction quality and negatively impact their preschool children’s behavioural outcomes, a finding that is supported by the existing literature [129,130,131]. Therefore, mothers who exhibit subclinical levels of depression should also be supported by healthcare professionals [130,131]. This may be of particular relevance to childhood externalizing problems, as they were associated with the subclinical depressive symptom profile, but not the high depressive symptom profile. Focusing solely on mothers with clinical levels of depressive symptoms could ultimately result in many at-risk mother–child dyads being overlooked and prevent or delay the delivery of interventions aimed at reducing the effects of suboptimal maternal mental health on children’s behavioural outcomes [130,131].

### 4.3. Child Sex-Assigned-at-Birth and Sociodemographics: Additional Child Behaviour Predictors

While some studies posit that male children are more likely to develop both types of behavioural problems [4,5,6,7,8], findings derived from other studies [60,61,62] suggest that female children are more likely to develop internalizing problems and male children are more likely to develop externalizing problems. Findings from our study partially support the latter idea; male children were more likely to exhibit externalizing problems. This may be attributable to prenatal conditions (e.g., greater levels of testosterone in male child pregnancies) [63,132,133], heritability [64,65], or brain maturation rates [60], but also to social forces, which reinforce gendered behaviours [124,134]. However, studies also show that parents are less likely to hold their children to gendered behavioural standards when mothers report high educational attainment [135]. As mothers in this study showed high level of educational attainment, our findings support the contention that biological forces, as opposed to social forces, may be more influential in the development of sex-specific child behavioural problems. Regardless, findings from this study demonstrate children’s sex-specific vulnerability to maternal depressive symptom profiles, helping to inform interventions that aim to prevent children’s behavioural problems.

Socioeconomic status was also negatively associated with externalizing problems, serving as a protective factor. This corroborates literature that indicates that children who reside in households with mothers who report higher educational attainment [136], higher annual household income [137], and being partnered [138] often experience more optimal parent–child interactions. On the other hand, gestational age was not associated with children’s behavioural problems, a finding inconsistent with what is known [77,78]. For example, children born extremely preterm (i.e., <32 weeks) are at a greater risk of experiencing development concerns [77,78]; therefore, it is more likely that the lack of variability in lower gestational age led to null findings vis-à-vis gestational age as a predictor. Further, maternal age was not significantly associated with preschool children’s behavioural problems despite evidence that older mothers tend to have more experience and stability, which can enhance parent–child interaction quality and their children’s behavioural outcomes [79]. However, due to the high socioeconomic homogeneity of this sample, younger mothers may have had access to resources such as parenting programs, which enhanced their skills in parent–child interactions [139]. Lastly, lower levels of maternal perceived social support were only associated with children’s internalizing problems, though the small estimate suggests low clinical relevance. Perhaps social support alleviates certain features of maternal depressive symptoms (e.g., loneliness and negative self-appraisals) [140], that in turn, could contribute to children’s internalizing problems [141].

### 4.4. Exploring Impacts of Maternal Depressive Symptoms on Child Behavioural Subscales

Post hoc exploration generally indicated that mothers’ subclinical depressive symptoms, but not high depressive symptoms, were associated with children’s behavioural problem subscale scores. For example, in the present study it was found that externalizing problems, including hyperactivity, aggression, and attention problems, were associated with the subclinical depressive symptom profile, but not the high depressive symptom profile. Further, children’s anxiety and depression, but not somatization, were also associated with the subclinical depressive symptom profile. Moreover, male children were more likely than female children to exhibit problems on the externalizing problems subscales, which aligns with anticipated findings that male children are more likely to exhibit various externalizing problems [60,61,62]. While it was hypothesized that high maternal depressive symptoms would be associated with children’s behavioural problems [142], our unexpected findings could indicate that mothers who experience subclinical symptoms may be less likely to be identified or seek help from support systems (e.g., family, friends, and providers) than mothers who experience high depressive symptoms [143]. This lack of support for their depressive symptoms could undermine parent–child interactions, which in turn, would negatively impact their children’s behavioural outcomes [15]. On the other hand, highly depressed mothers may be more likely to be referred to or provided with parenting support [143], and so, while their depressive symptomology may remain elevated, their parent–child interaction quality may be enhanced, contributing to more optimal child behavioural development [125]. This reinforces that diagnostic levels of mothers’ depressive symptoms may not always be a definitive predictor of children’s behavioural health and that women with subclinical levels of depressive symptoms should be targeted to prevent intergenerational impacts on child development.

### 4.5. Limitations and Strengths

A limitation of this study is that most participants reported high socioeconomic attainment, which was likely exacerbated by selective attrition bias, decreasing the diversity of participants and the study’s generalization to other groups [144]. While findings may be generalizable to groups with similar demographic and sociopolitical conditions, more research is needed before generalizing the findings across different cultural groups. In addition, fathers’ depressive symptoms were not included in the analysis, a factor that is relevant to children’s behavioural outcomes [20]. Nevertheless, many factors can contribute to children’s behaviour problems, and this study controlled for many of them, which is a strength [14]. Although many researchers have found it useful to undertake latent profile analysis of depression [50,51,52,53], some may argue that this approach is overly reductive. Other contextual factors could have been considered, such as comorbidities and diagnoses [145,146]; however, these data were not available to us in sufficient sample sizes. Future research should consider these factors. Self-report instruments, such as the EPDS used to measure depressive symptoms, can result in bias, especially in mental health research where individuals may be more likely to underreport their condition or answer based on their current emotional state [147]. Nevertheless, the measures employed in this study were psychometrically valid and reliable measures, decreasing the effect of self-report bias on the internal validity of this study. This study is strengthened by its longitudinal study design, which established a clear exposure–outcome relationship using maternal depressive symptom data collected across numerous prenatal, postpartum, and early childhood time points, which were used to best group mothers’ depressive symptoms.

## 5. Conclusions

This longitudinal study employed latent profile analysis to identify profiles that most adequately described mothers’ depressive symptom trajectories across prenatal and postpartum periods and explored these profiles as predictors of their preschool children’s behavioural problems. As hypothesized, a three-profile structure, composed of minimal, subclinical, and high depressive symptoms, was deemed the most suitable and parsimonious for characterizing mothers’ depressive symptom trajectories, using depressive symptoms measured at early and late pregnancy and at 3, 6, 12, 24, and 36 months postpartum. Findings showed that subclinical levels of depressive symptoms in mothers were associated with their preschool children’s behavioural outcomes, whereas high levels of depressive symptoms were associated with their children’s internalizing problems, emphasizing the need to support mothers exhibiting both subclinical and high levels of depressive symptoms to prevent the potential detrimental effects of suboptimal maternal mental health on children’s behavioural outcomes. Targeting mothers’ depressive symptoms could minimize the likelihood of behavioural problems emerging in their preschool children, prevent negative long-term negative outcomes, and promote lifelong quality of life.

## Figures and Tables

**Table 1:** Sample characteristics (n = 704).

Variables	Frequency (n, %)	Mean (SD)	Min, Max
Sociodemographic Variables			
*Child Sex-Assigned-at-Birth*			
Female	346 (49)		
Male	358 (51)		
*Social–Economic Status*			
0	5 (1)		
1	50 (7)		
2	163 (23)		
3	477 (68)		
Missing	9 (1)		
*Gestational Age at Birth (weeks)*		39.3 (1.6)	31.7, 42.0
Missing	3 (0)		
*Childbirth Weight (grams)*		3378.0 (515.5)	1380, 4900
Missing	4 (1)		
*Maternal Age (years)*		32.2 (4.0)	20.3, 44.3
*Maternal Social Support*		14.1 (2.7)	1, 16
Missing	74 (11)		
Predictor Variable			
*Maternal Depression Profiles*			
Minimal depressive symptoms Subclinical symptoms High depressive symptoms	361 (51) 278 (39) 65 (9)		
Outcome			
*Behavior Assessment System for Children Internalizing (T-score)*		48.8 (8.8)	27, 78
*Behavior Assessment System for Children Externalizing (T-score)*		48.3 (8.0)	31, 85

**Table 2:** Model fit indices for latent profiles of depression symptoms from pregnancy to three years postpartum (n = 704).

Model	Likelihood Ratio Statistic	Bayesian Information Criterion	Akaike Information Criterion	Entropy	Vuong-Lo-Mendell-Rubin	*p*-Value
1-profile	−12,207.93	24,443.88	24,507.67	-	-	-
2-profile	−11,490.94	23,025.90	23,126.15	0.86	1 vs. 2 profiles	0.000
3-profile	−11,233.37	22,526.76	22,663.46	0.88	2 vs. 3 profiles	0.000
4-profile	−11,131.19	22,338.40	22,511.55	0.86	3 vs. 4 profiles	0.526

**Table 3:** General linear regression model predicting children’s internalizing problems at 5 years of age.

Variable	T-Score Estimates (SE)	95% Confidence Interval	*p*-Value
Unadjusted Analysis			
*Intercept*	47.01 (0.45)	46.12, 47.89	***<0.001***
*Depression Profile Reference (Minimal)*	**-**	**-**	**-**
*Subclinical*	3.73 (0.69)	2.39, 5.08	***<0.001***
*High*	3.78 (1.16)	1.51, 6.05	***0.002***
Adjusted Analysis			
*Intercept*	54.17 (9.24)	6.02, 72.32	***<0.001***
*Female*	1.19 (0.66)	−0.10, 2.50	0.071
*Depression Profile Reference (Minimal)*	**-**	**-**	**-**
*Subclinical*	3.01 (0.71)	1.60, 4.43	***<0.001***
*High*	2.81 (1.22)	0.39, 5.22	***0.022***
*Socioeconomic Status*	−0.67 (0.54)	−1.73, 0.38	0.209
*Gestational Age at Birth (weeks)*	0.03 (0.21)	−0.38, 0.45	0.874
*Maternal Age (years)*	−0.06 (0.08)	−0.24, 0.10	0.461
*Maternal Social Support At 3 Years*	−0.35 (0.12)	−0.60, −0.10	***0.005***
Post Hoc Analysis (ANOVA)			
*Subclinical versus Minimal*		3.73 (2.12, 5.34)	***0.000***
*High versus Minimal*		3.77 (1.06, 6.49)	***0.003***
*High versus Subclinical*		0.05 (−2.73, 2.82)	0.999

NOTE: SE = standard error; bolded *p*-values are statistically significant.

**Table 4:** General linear regression model predicting children’s externalizing problems at 5 years of age.

Variable	T-Score Estimates (SE)	95% Confidence Interval	*p*-Value
Unadjusted Analysis			
*Intercept*	47.14 (0.42)	46.32, 47.96	***<0.001***
*Depression Profile Reference (Minimal)*	**-**	-	**-**
*Subclinical*	2.42 (0.63)	1.18, 3.67	***<0.001***
*High*	1.67 (1.07)	−0.43, 3.77	0.118
Adjusted Analysis			
*Intercept*	56.52 (8.79)	39.25, 73.79	***<0.001***
*Female*	−2.16 (0.63)	−3.41, −0.92	***0.001***
*Depression Profile Reference (Minimal)*	**-**	**-**	**-**
*Subclinical*	2.20 (0.68)	0.86, 3.54	***0.001***
*High*	1.01 (1.16)	−1.28, 3.30	0.388
*Socioeconomic Status*	−1.63 (0.51)	−2.64, −0.62	***0.001***
*Gestational Age at Birth (weeks)*	−0.08 (0.20)	−0.48, 0.30	0.664
*Maternal Age (years)*	0.01 (0.08)	−0.15, 0.17	0.897
*Maternal Social Support At 3 Years*	−0.06 (0.12)	−0.30, 0.17	0.583
Post Hoc Analysis (ANOVA)			
*Subclinical versus Minimal*		2.42 (0.93, 3.91)	***0.000***
*High versus Minimal*		1.67 (−0.83, 4.18)	0.262
*High versus Subclinical*		−0.75 (−3.32,1.81)	0.770

NOTE: SE = standard error; bolded *p*-values are statistically significant.

**Table 5:** Summary of estimates and *p*-values from post hoc exploration of each child behavioural problem subscale.

Variable	Externalizing Problems			Internalizing Problems		
	Hyperactivity	Aggression	Attention	Anxiety	Depression	Somatization
Unadjusted Analysis (T-score estimates (SE))						
*Intercept*	***47.43 (0.43)*** **	***47.33 (0.41)*** **	***47.51 (0.39)*** **	***49.33 (0.49)*** **	***48.83 (0.46)*** **	***45.02 (0.42)*** **
*Depression Profile* *Reference (Minimal)*	-	-	-	-	-	-
*Subclinical*	***2.93 (0.66)*** **	***1.53 (0.62)*** *	***2.31 (0.60)*** *	***3.95 (0.75)*** **	***2.90 (0.70)*** **	***1.70 (0.63)*** *
*High*	1.70 (1.12)	1.26 (1.06)	1.01 (1.0)	***5.77 (1.27)*** **	2.19 (1.19)	0.75 (1.07)
Adjusted Analysis (T-score estimates (SE))						
*Intercept*	***63.00 (9.27)*** **	***49.08 (8.67)*** **	***69.62 (8.21)*** **	***45.60 (10.3)*** **	***54.90 (9.86)*** **	60.39 (8.50)
*Female*	***−2.47 (0.66)*** *	***−1.53 (0.62)*** *	***−2.08 (0.59)*** *	0.69 (0.74)	0.81 (0.70)	1.21 (0.61)
*Depression Profile* *Reference (Minimal)*	-	-	-	-	-	-
*Subclinical*	***2.68 (0.72)*** *	***1.39 (0.67)*** *	***1.68 (0.63)*** *	***3.48 (0.80)*** **	***2.40 (0.76)*** *	1.03 (0.66)
*High*	0.99 (1.23)	0.75 (1.15)	−0.51 (1.09)	***5.03 (1.36)*** **	1.61 (1.30)	−0.13 (1.12)
*Social Economic Status*	***−1.73 (0.54)*** *	***−1.24 (0.50)*** *	***−2.11 (0.48)*** **	−0.41 (0.60)	−0.61 (0.57)	0.51 (0.49)
*Gestational Age at Birth (weeks)*	−0.25 (0.21)	0.09 (0.19)	−0.28 (0.18)	0.28 (0.23)	0.03(0.22)	−0.26 (0.19)
*Maternal Age (years)*	0.03 (0.08)	−0.01 (0.08)	−0.02 (0.07)	−0.04 (0.09)	−0.11 (0.09)	−0.01 (0.08)
*Maternal Social Support At 3 Years*	−0.06 (0.12)	−0.06 (0.11)	***−0.23 (0.11)*** *	***−0.37(0.14)*** *	−0.16 (0.13)	−0.25 (0.11)

NOTE: ** *p* ≤ 0.001, * *p* < 0.05; SE = standard error; bolded estimates are statistically significant.

## Data Availability

The original contributions presented in the study are included in the article, further inquiries can be directed to the corresponding author.

## Supplementary Materials

The following supporting information can be downloaded at: https://www.mdpi.com/article/10.3390/children12050535/s1, Table S1: Guidelines for Reporting on Latent Trajectory Studies.
