# Supplementary material for: Are Children’s Externalizing and Internalizing Behaviours at 5 Years Predicted by Maternal Perinatal Depression Trajectory Profiles?
Source: Children (Basel). 2025 Apr 23;12(5):535. doi: 10.3390/children12050535 (PMC12110483; doi:10.3390/children12050535)
Supplement: Supplementary file 1 [file children-12-00535-s001.zip › children-3584740-supplementary.pdf]

Table S1. Guidelines for Reporting on Latent Trajectory Studies

Item Number:	Checklist Item:	Page Number:
1.	Is the metric of time used in the statistical model reported?	10 & 17
2.	Is information presented about the mean and variance of time within a wave	13
3a.	Is the missing data mechanism reported?	15
3b.	Is a description provided of what variables are related to attrition/missing data?	13
3c.	Is a description provided of how missing data in the analyses were dealt with?	13
4.	Is information about the distribution of the observed variables included?	14
5.	Is the software mentioned?	13
6a.	Are alternative specifications of within-class heterogeneity considered (e.g., LGCA vs. LGMM) and clearly documented? If not, was sufficient justification provided as to eliminate certain specifications from consideration?	26
6b.	Are alternative specifications of the between-class differences in variance–covariance matrix structure considered and clearly documented? If not, was sufficient justification provided as to eliminate certain specifications from consideration?	11-14
7.	Are alternative shape/functional forms of the trajectories described?	16
8.	If covariates have been used, can analyses still be replicated?	22
9.	Is information reported about the number of random start values and final iterations included?	Non-relevant
10.	Are the model comparison (and selection) tools described from a statistical perspective?	5
11.	Are the total number of fitted models reported, including a one-class solution?	12-13
12.	Are the number of cases per class reported for each model (absolute sample size, or proportion)?	15
13.	If classification of cases in a trajectory is the goal, is entropy reported?	16
14a.	Is a plot included with the estimated mean trajectories of the final solution?	15
14b.	Are plots included with the estimated mean trajectories for each model?	17
14c.	Is a plot included of the combination of estimated means of the final model and the observed individual trajectories split out for each latent class?	17
15.	Are characteristics of the final class solution numerically described (i.e., means, SD/SE, n, CI, etc.)?	18

16.	Are the syntax files available (either in the appendix, supplementary materials, or from the authors)?	29
